# Autosomal Recessive Nonsyndromic Hearing Impairment due to a Novel Deletion in the *RDX* Gene

**DOI:** 10.4061/2011/294675

**Published:** 2011-11-01

**Authors:** Kwanghyuk Lee, Mohammad Amin ud Din, Muhammad Ansar, Regie Lyn P. Santos-Cortez, Wasim Ahmad, Suzanne M. Leal

**Affiliations:** ^1^Department of Molecular and Human Genetics, Baylor College of Medicine, One Baylor Plaza, Houston, TX 77030, USA; ^2^Department of Biology, Government Degree College, Dera Ghazi Khan 32200, Pakistan; ^3^Department of Biochemistry, Faculty of Biological Sciences, Quaid-I-Azam University, Islamabad 45320, Pakistan

## Abstract

The *RDX* gene anchors cytoskeletal actin of stereocilia to hair cell transmembrane and is responsible for autosomal recessive nonsyndromic hearing impairment (ARNSHI) due to DFNB24. A genome scan was performed using DNA samples from a consanguineous Pakistani family with ARNSHI. A significant maximum two-point LOD score of 4.5 (*θ* = 0) and multipoint LOD score of 5.8 were achieved at marker D11S1998 (chr11 : 117.20 Mb). The region of homozygosity is bounded by markers D11S2000 (105.06 Mb) and D11S4464 (123.13 Mb) and contains the NSHI genes *TECTA* and *RDX*. Although no potentially causal variants were identified in the *TECTA* gene, within the *RDX* gene a novel deletion c.1076_1079delTTAA (p.Ile359Lysfs∗6) was identified. The *RDX* deletion segregates with ARNSHI within the family and was not observed in 500 control chromosomes. It is predicted to cause premature truncation of radixin at the **α**-helical domain and to result in nonfunctional transcripts within the cochlea. *RDX* isoforms which encode the coiled-coil region of the **α**-helical domain are deemed necessary for proper function of hair cell stereocilia.

## 1. Introduction

Within the mammalian cochlea, the most specialized cells for hearing that mediate conversion of mechanical vibration from sound energy to neural impulses in the brain (mechanotransduction) are the hair cells, so-named because of the hair-like projections at their apical surfaces, which are called stereocilia. Of the 54 nonsyndromic hearing impairment (NSHI) genes that are currently known, *∼*20 genes are associated with stereocilia structure or assembly (Hereditary Hearing Loss Homepage). Variants in *RDX* (MIM 179410) cause autosomal recessive (AR) NSHI (DFNB24) and can be found in all three domains of the radixin protein [1, 2]. Radixin is a member of the ERM family of highly homologous proteins including ezrin and moesin. ERM proteins link cell membrane proteins to actin, which is the basic component of the cytoskeleton of microvillar structures such as stereocilia [3]. There are three well-characterized ERM protein domains: (1) the FERM domain, which binds to transmembrane proteins [4]; (2) the *α*-helical domain, which masks interaction sites of the FERM domain to regulate activation [5]; (3) the C-terminal tail or ERM association domain (C-ERMAD) which has an F-actin-binding site [6]. Consistent with its anchoring function in the hair cell, radixin is mainly located at the stereociliary base [7]. This paper describes the fifth family known to date to have ARNSHI due to an *RDX *mutation. 

## 2. Materials and Methods

The study was approved by the Institutional Review Boards of Quaid-I-Azam University and the Baylor College of Medicine and Affiliated Hospitals. Informed consent was obtained from all members of family 4208 who participated in the study. 

**Figure 1:**
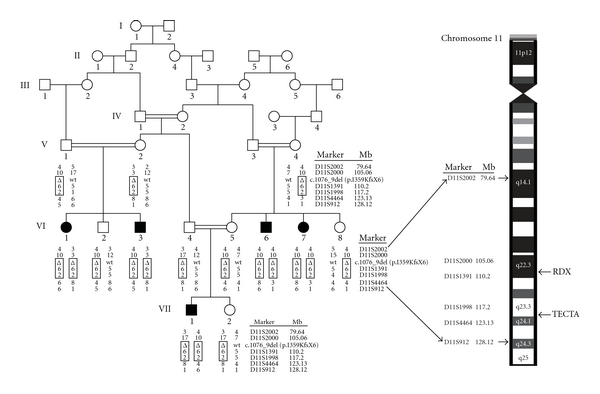
Pedigree drawing and haplotype of family 4208 with the genetic interval on chromosome 11. *Filled *symbols denote individuals with ARNSHI, while *clear* symbols represent hearing individuals. The haplotype segregating with ARNSHI is shown in a *box *and includes short tandem repeat markers and the *RDX *deletion c.1076_1079delTTAA (p.Ile359Lysfs*6) as a *triangle.* The positions of the *TECTA *and* RDX *genes relative to the genome scan markers are labelled. The genetic interval with genotyped marker loci and locations of *RDX* and *TECTA* genes were indicated on the cartoon of chromosome 11.

The members of family 4208 are from the Muzafar Ghar district of Punjab province. Venous blood was obtained from 13 members of family 4208, five of whom have HI (Figure 1). Genomic DNA was extracted from whole blood following a standard protocol [8]. All 13 samples underwent a whole genome linkage scan at the Center for Inherited Disease Research (CIDR) using 405 short tandem repeat markers with average spacing of 9 cM. Data quality control was performed on the resulting genotype data using PedCheck [9] in order to identify Mendelian inconsistencies and MERLIN [10] to detect occurrences of double recombination events over short genetic distances, which are most likely due to genotyping error. Two-point linkage analysis was performed with MLINK of the FASTLINK package [11]. Multipoint linkage analysis was carried out using Allegro1.2c [12]. An AR mode of inheritance with complete penetrance and a disease allele frequency of 0.001 were used in the parametric linkage analysis. Marker allele frequencies were estimated from observed and reconstructed genotypes of founders from family 4208 and 35 other families who underwent genome scan at the same time at CIDR. For multipoint linkage analysis, genetic map positions were based on the Rutgers combined linkage-physical map of the human genome Build 36 version [13]. For markers used in the analysis which are not included on the Rutgers map, the physical map position from the human reference sequence (Build 36) was used to interpolate the genetic map position on the Rutgers map. Haplotypes were reconstructed using SimWalk2 [14]. 

All exons of the *RDX *(MIM 179410; NM_002906.3) and *α*-tectorin (*TECTA *[MIM 602574; NM_005422.2]) genes were sequenced in HI individuals VI-1 and VII-1 and hearing individual VI-5 who are members of family 4208 (Figure 1). After PCR amplification and purification, sequencing was performed using the BigDye Terminator v3.1 Cycle Sequencing Kit and the ABI 3730 DNA Analyzer (Applied Biosystems Inc., Foster City, Calif, USA). The resulting sequences were assembled and analysed using the Sequencher software (Gene Codes Corp., Ann Arbor Mizh, USA). After identification of the *RDX *deletion, exon 10 was sequenced using DNA samples from additional family members and 250 unrelated hearing control individuals from Pakistan.

## 3. Results and Discussion

Family 4208 (Figure 1) is a consanguineous kindred with ARNSHI from the Punjab region of Pakistan and the family members speak Saraiki. The hearing impairment (HI) was described by family members as prelingual in onset, bilateral, and profound. No possible environmental causes of HI such as perinatal, ototoxic, traumatic, and infectious factors were elicited from the clinical history. Evidence of syndromic and vestibular phenotypes was not found after physical examination that included balance and gait testing. 

A significant maximum two-point LOD score of 4.5 was obtained at marker D11S1998 (chr11 : 117.20 Mb) at *θ* = 0. At the same marker, a maximum multipoint LOD score of 5.8 was obtained. The 3-unit support interval (Table 1) and the region of homozygosity (Figure 1) completely overlap, with the proximal limit at D11S2000 (105.06 Mb) and the distal limit at D11S4464 (123.13 Mb). The linkage interval extends over a 25.28 cM region at 11q22.3-q24.1 and contains 18.07 Mb of sequence.

**Figure 2:**
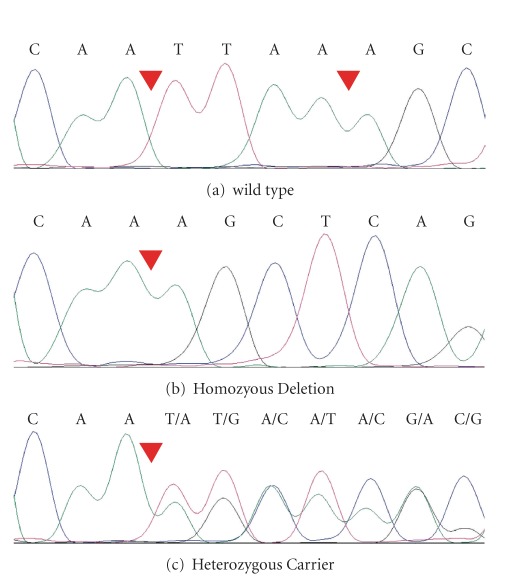
Chromatogram displaying the novel *RDX* deletion c.1076_1079delTTAA (p.Ile359Lysfs*6). Chromatograms shown are from sequences of the following: (a) wild type, (b) hearing-impaired homozygous individual VII-1, and (c) unaffected heterozygous individual VI-5. The deleted nucleotides TTAA are indicated with a red triangle.

**Figure 3:**
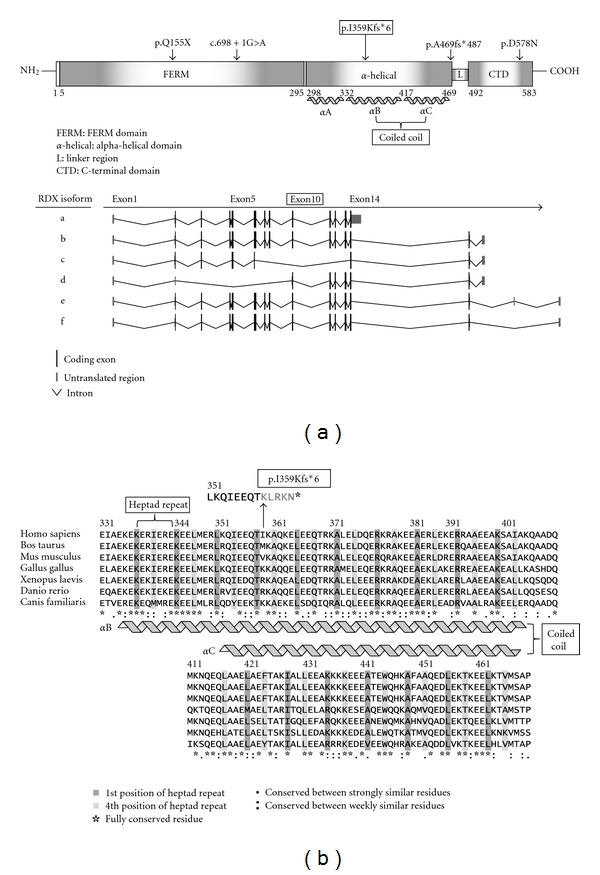
(a) Schematic representation of protein domains and isoforms of *RDX* gene. The positions of known pathogenic mutations were indicated with arrow and the newly identified mutation was boxed. The protein domain structure is based on the reference sequence NP_002897 (transcript isoform a). (b) Clustal W multiple sequence alignment of alpha-helical domain of *RDX* protein sequences of seven different species. The 1st and 4th amino acid residues of heptad repeat sequences are highly conserved through the species.

Within the linkage interval there are 167 RefSeq genes, including two NSHI genes, *RDX *and *TECTA. *Both genes were sequenced. No sequence variants were identified in the *TECTA *gene. On the other hand, a novel *RDX *deletion c.1076_1079delTTAA (p.Ile359Lysfs*6), which segregates with ARNSHI in family 4208 (Figure 2), was identified. This deletion was not found in 500 control chromosomes, in either the homozygous or in the heterozygous state. The deletion results in a frameshift and premature truncation of the radixin protein to 363 residues and, subsequently, in loss of most of the *α*-helical domain and the whole F-actin-binding domain (Figure 3(a)). According to the PROSITE database [15], the deleted domains contain multiple phosphorylation sites for protein kinase C and casein kinase II, which are receptor specific for the ERM protein family [16]. 

The *TECTA* gene causes both autosomal dominant (DFNA8/12) and AR (DFNB21) NSHI [17]. When inherited recessively, *TECTA* mutations cause moderate-to-severe hearing impairment with a flat or U-shaped audiogram pattern [18]. In contrast, the HI in family 4208 is profound, which is similar to the previously reported HI pattern for *RDX* [2]. In *Rdx* ^−/−^ mice, degeneration of outer hair cell stereocilia began at onset of hearing [19], and this is consistent with the prelingual onset of HI in humans with *RDX *mutations, as was observed in family 4208. Congenital jaundice was also noted in *Rdx* ^−/−^ mice [20]. However there is no evidence of hyperbilirubinemia in family 4208. Additionally in the previously reported four families that segregate *RDX *mutations there is no evidence of hyperbilirubinemia [1, 2]. 

The mutation c.1076_1079delTTAA is located in exon 10 of *RDX. *Of the six isoforms of *RDX* (Figure 3(a)), isoform c does not include exons 7 to 13 of isoform a. In the original DFNB24 family, the p.Gln155* stop codon mutation can be found in exon 5, which is not included in *RDX* isoform d [2] (Figure 3(a)). In particular, exon 10 marks the beginning of a series of highly conserved heptad repeats in the *α*B helix which interacts with the corresponding highly conserved heptad repeats in the *α*C helix (Figure 3(b)) as the *α*-helical domain folds on itself to form an antiparallel coiled coil in the dormant state [5] (Figures 3(a) and 3(b)). When active, the coiled coil is fully extended, unmasking ligand-binding sites [5]. This may indicate that the *α*B/*α*C coiled coil is necessary for regulation of radixin activity within hair cell stereocilia. 

## 4. Conclusion

A novel deletion c.1076_1079delTTAA (p.Ile359Lysfs*6) in the *RDX *gene was identified in a large Pakistani consanguineous pedigree that segregates ARNSHI. This novel *RDX *deletion is predicted to cause premature truncation of radixin at the *α*-helical domain and to result in nonfunctional transcripts within the cochlea. Identification of the c.1076_1079delTTAA (p.Ile359Lysfs*6) deletion gives a better understanding of the role the *RDX* gene plays in hearing impairment.

##  Electronic Database Information

The following URLs were accessed for data in this paper: Hereditary Hearing Loss Homepage (http://hereditaryhearingloss.org/); UCSC Genome Browser (http://genome.ucsc.edu/); OMIM (http://www.omim.org/). ClustalW (http://www.ebi.ac.uk/Tools/msa/clustalw2/).

## Figures and Tables

**Table 1:** Two-point and multipoint LOD scores for family 4208 at chromosome 11q22.3-q24.1.

Marker name^1^	Physical map position^2^	Genetic map position^3^	Multipoint LOD score	Two-point LOD score at *θ* =					
				0.0	0.01	0.05	0.10	0.20	0.30
D11S2371	73,182,778	84.41	−∞	−∞	−0.35	0.24	0.37	0.31	0.15
D11S2002	79,643,050	91.48	−13.66	−∞	−1.41	−0.18	0.20	0.33	0.22
**D11S2000**	**105,063,887**	**111.71**	−6.18	−6.08	−0.15	0.90	1.07	0.83	0.42
D11S1391	110,200,327	115.14	5.20	3.18	3.10	2.74	2.29	1.43	0.68
D11S1998	117,202,969	126.24	5.78	4.47	4.36	3.95	3.41	2.33	1.26
**D11S4464**	**123,131,592**	**136.99**	−∞	−∞	−2.84	−0.93	−0.25	0.16	0.17
D11S912	128,129,301	145.13	−∞	−∞	−1.52	−0.62	−0.25	0.01	0.06
D11S968	133,323,584	160.10	−4.03	−1.95	−0.06	0.54	0.64	0.45	0.18

^1^Markers in bold denote marker limits based on the 3-unit support interval and the homozygous region.

^2^Physical map positions in base pairs from Build 36 of the human reference sequence.

^3^Genetic map positions in cM from Rutgers combined linkage-physical map of the human genome Build 36 version.
